# Sex and genetic differences in postoperative cognitive dysfunction: a longitudinal cohort analysis

**DOI:** 10.1186/s13293-019-0228-8

**Published:** 2019-03-29

**Authors:** Katie J. Schenning, Charles F. Murchison, Nora C. Mattek, Jeffrey A. Kaye, Joseph F. Quinn

**Affiliations:** 1 Department of Anesthesiology and Perioperative Medicine, Oregon Health & Science University, Mail Code L459, 3181 SW Sam Jackson Park Rd, Portland, OR 97239 USA; 2 Department of Neurology, Oregon Health & Science University, Portland, OR 97239 USA; 3 Oregon Center for Aging and Technology, Oregon Health & Science University, Portland, OR 97239 USA; 4 Department of Neurology, Portland Veterans Affairs Medical Center, Portland, OR 97239 USA; 5 Layton Aging and Alzheimer’s Disease Center, Oregon Health & Science University, Portland, OR 97239 USA; 6 Department of Biostatistics, University of Alabama at Birmingham, Birmingham, AL 97239 USA

**Keywords:** Epidemiology, Anesthesia, Surgery, Cognitive decline, Postoperative, Apolipoprotein E ε4 (*APOE4*), Alzheimer’s disease, Cohort study, Sex influence

## Abstract

**Background:**

Postoperative cognitive dysfunction (POCD) is a common postoperative complication experienced by patients aged 65 years and older, and these older adults comprise more than one third of the surgical patients in the USA. Because not everyone with a history of exposure to surgery and anesthesia develops POCD, there are likely major biological risk factors involved. There are important gaps in our knowledge regarding whether genetic makeup, biological sex, or other Alzheimer’s disease risk factors predispose older adults to developing POCD. We set out to determine whether biological sex and Apolipoprotein E-ε4 (*APOE4*) carrier status increase the risk of developing POCD in older adults.

**Methods:**

We performed a cohort analysis of 1033 participants of prospective longitudinal aging studies. Participants underwent regular cognitive test batteries and we compared the annual rate of change over time in various cognitive measures in the women exposed to surgery and general anesthesia compared to the men exposed to surgery and general anesthesia. Mixed-effects statistical models were used to assess the relationship between biological sex, *APOE4* carrier status, surgery and anesthesia exposure, and the rate of change in cognitive test scores.

**Results:**

When comparing all men (*n* = 89) and women (*n* = 164) who had surgery, there were no significant sex differences in postoperative cognitive outcomes. However, men with an *APOE4* allele performed significantly worse on cognitive testing following surgery and anesthesia than women *APOE4* carriers, even after adjusting for age, education level, and comorbidities.

**Conclusions:**

Older men with *APOE4* allele may be more vulnerable to postoperative cognitive dysfunction than older women with *APOE4* allele.

## Background

Adults 65 years and older represent the fastest-growing age group in the USA and account for one third of all surgical patients. These older adults are at the highest risk for deleterious perioperative neurocognitive disorders (PND) such as postoperative delirium and postoperative cognitive dysfunction (POCD). Postoperative delirium occurs in up to 65% of older adults and is characterized by inattention and confusion [1]. POCD, occurring in up to 40% of older surgical patients [2], is a syndrome characterized by an objective decline in cognition postoperatively when compared to preoperative function as measured by a decrease in performance on a neuropsychological test battery. POCD is independently associated with increased mortality, length of hospitalization, healthcare costs, early departure from the workforce, and overall decreased quality of life [2, 3]. Although postoperative delirium and POCD are separate entities, it is possible that they are interrelated. Studies show that patients who experience postoperative delirium are at increased risk for POCD and are even at a greater risk of progression to dementia [4, 5]. However, because not every older adult exposed to surgery and anesthesia develops PND, there are likely biological risk factors involved. While there is considerable evidence to support age, education level, and preexisting cognitive dysfunction as risk factors for PND, it is currently unclear whether the interaction of biological sex or genetic risk factors with exposure to surgery and anesthesia leads to either postoperative delirium or POCD [6].

Several recent large epidemiologic studies found that anesthesia and surgery not only lead to postoperative cognitive dysfunction (POCD) but also increase the Alzheimer’s disease (AD) risk [1, 7]. Similarly, a prospective longitudinal study of older men and women undergoing cardiac surgery found that the prevalence of dementia 7.5 years after cardiac surgery was greatly increased compared to population prevalence [4]. Human biomarker investigations reveal that ratios of phosphorylated tau:amyloid beta (Aβ) in the cerebrospinal fluid convert to an AD pattern postoperatively [5, 8] and that surgical patients have increased rates of brain atrophy compared to controls [9, 10]. Further, animal studies indicate that surgery and anesthesia contribute to cognitive decline and AD-associated neuropathologic changes including neuroinflammation, Aβ aggregation, and tau hyperphosphorylation [8, 11–15].

It is well established that the gene most strongly associated with AD is apolipoprotein E (*APOE*). However, to date, there are conflicting reports in the literature regarding whether the presence of an *APOE4* allele confers a higher risk for developing postoperative cognitive dysfunction (POCD) [16]. Further, it is known that the *APOE4* allele confers an increased risk of AD in a sex-dependent manner, disproportionately affecting women in both prevalence and severity [17]; almost 2/3 of American seniors living with AD are women. On the other hand, several studies have shown that older men are more likely to develop postoperative delirium when compared to older women [18–20].

In order to begin to elucidate the role of genetic factors and biological sex in the development of POCD, we investigated the associations of sex, *APOE4* carrier status, and exposure to surgery/general anesthesia (GA) with postoperative cognitive decline in 1033 participants from several prospective longitudinal cohort studies of the Oregon Alzheimer’s Disease Center (OADC). We hypothesized that *APOE4*+ women and *APOE4*+ men exposed to surgery and general anesthesia would experience different rates of postoperative cognitive decline.

## Methods

### Database sources and study population

We performed a retrospective cohort analysis of 1033 subjects who participated in a number of Oregon Alzheimer’s disease Center (OADC) longitudinal cohort studies: the Oregon Brain Aging Study (OBAS) (*n* = 297) [21, 22], the Intelligent Systems for Assessment of Aging Changes (ISAAC) Study (*n* = 116) [23], the Klamath Exceptional Aging Project (KEAP) (*n* = 175) [24, 25], the African American Dementia and Aging Project (AADAPt) (*n* = 54), the Oregon Community Brain Donor Program (CBDP) (*n* = 97), the Oregon Living Laboratory (OLL) (*n* = 59), and the Layton Alzheimer’s Disease Center patient registry (*n* = 235). All studies were approved by the Oregon Health & Science University’s institutional review board, and all participants provided written informed consent. Using the above cohorts, we derived a comprehensive longitudinal dataset including demographic background, known *APOE* status, (determined via restriction digest, sequencing a PCR product, or by SNP genotyping) and cognitive decline. In order to appropriately contribute to the longitudinal analyses, all participants were required to have at least one follow-up visit during their study involvement for inclusion into the present cohort. At time zero (study enrollment), all participants began in the non-surgical group. Participants were then assigned to the surgical group upon undergoing surgery/GA. We compared the rate of change over time in various cognitive measures (Table 1). Participants underwent cognitive test batteries annually.

**Table 1:** Outcome measures

Cognitive domain	Specific tests
Global cognitive function tests	Mini-Mental State Examination (MMSE), Clinical Dementia Rating (CDR), CDR sum of boxes (CDR-SB)
Executive function	Animal Category Fluency, Trail Making Test B
Attention & concentration	Symbol Digit Modalities Test (SDMT), Digit Symbol Test
Memory	Logical Memory Delayed Recall, Consortium to Establish a Registry for AD (CERAD) Word List Delayed Recall

### Outcome measures

Dementia assessments included the Mini-Mental State Examination (MMSE), Clinical Dementia Rating (CDR), and CDR sum of boxes. The battery of tests used in the neuropsychological analysis, which have been described previously [11], assessed multiple cognitive domains and included Animal Category Fluency and Trail Making Test B for executive function, the Symbol Digit Modalities Test (SDMT) and Digit Symbol Test for attention and concentration, and Logical Memory Delayed Recall and Consortium to Establish a Registry for AD (CERAD) Word List Delayed Recall for memory. The cognitive tests administered are standard, validated measures commonly used in longitudinal aging and dementia studies. The Clinical Dementia Rating is a scale used to stage dementia based on six domains of Memory, Orientation, Judgment and Problem-solving, Community Affairs, Home and Hobbies, and Personal Care. Each domain is rated on a five-point scale. The CDR sum of boxes is the total sum of these six points [26]. Mini-Mental State Examination (MMSE) is a 30-point questionnaire used to measure cognitive impairment and screen for dementia [27]. Animal Fluency is the number of animals a subject can name in 1 min [28]. Trail Making Test Part B requires the subject to draw lines between alternating numbers and letters in sequence as fast as they can. The score is the number of seconds to complete the test up to a maximum of 300 s [29]. Symbol Digit Modalities Test (SDMT) asks subjects to substitute numbers for corresponding symbols. Digit Symbol Test asks subjects to follow a key and draw the symbol that belongs with each number displayed in a series of rows. The score is the number of correct symbols drawn in 90 s [30]. Logical Memory Delayed Recall is a story memory test with a 20–30 min delay [31]. CERAD Word List Delayed Recall is a memory test. Ten words are presented and the subject is asked to recall the words again after a delay with distraction [32].

### Statistical analysis

Mixed-effects longitudinal regression was used to assess the influence of exposure to surgery/GA on the longitudinal change of the previously described response variables and how those alterations contrasted according to sex and *APOE4* carrier status. Main effects of surgery, sex, and *APOE4* carrier status on rates of change were first considered for subjects before assessment of the interactions between those key study covariates. Confounders previously identified as influences on the outcomes were selected a priori (age, education level, Cumulative Illness Rating Score (CIRS) at baseline) and were controlled for in all regression models; additionally, baseline values of outcomes were also controlled for when directly assessing rates of change. The Cumulative Illness Rating Score (CIRS) is a validated and reliable method to measure comorbidity previously cited as a key source of variance when evaluating these cohorts [33–35]. The key study covariates of interest, exposure to surgery/GA, *APOE4* status, and sex, were dichotomized to give four groups for each sex (Surgery-exposed *APOE4* carriers, unexposed *APOE4* carriers, exposed non-carriers, and unexposed non-carriers). Differences in the longitudinal rates of change were then directly contrasted between the groups using the appropriate design matrices to identify the corresponding effects of sex, genotype, and surgical exposure.

Carrier status of the *APOE4* allele pooled both heterozygous (*APOE2*,*4* and *APOE3*,*4)* and homozygous (*APOE4*,*4*) carriers into a single *APOE4+* group to create the dichotomous variable of *APOE4* carriers vs non-carriers. This was done due to the limited number of *APOE2*,*4* carriers and homozygous *APOE4*,*4* carriers as to avoid overfitting of model covariates. There were only three participants with homozygosity for E4 with surgical exposure; two men and one woman. In addition, no difference in homozygous vs heterozygous carriers was observed with respect to either sex or surgical exposure.

With multiple distinct longitudinal cohorts used in this study (i.e. OBAS, ISAAC, etc.), the annual rates of change in outcomes were contrasted among the different studies to evaluate any potential cohort dependencies. Cohort-based population differences were observed in the response at entry into the studies but had no influence on rates of change, the principal consideration of the current study, and were therefore considered differences in natural history for the subjects. With no observable difference in the time-dependent changes in outcomes between the study populations, the multiple cohorts were pooled into a single disposition.

A mixed-effects model framework was used to most appropriately leverage the repeated visits within the studies and correct for serial correlation according to subject. A compound symmetry error covariance model was used with parameters estimated using restricted maximum likelihood procedures with missing data points in the analytical sample considered missing at random. Model diagnostics used a combination of formal fit criteria, specifically Cook’s distance and the observation leverages, and visual inspection of the residual plots to evaluate model fit and integrity. Individual data points within subjects found to be both outcome outliers as well as having undue and extensive influence on sensitivity of longitudinal change were removed from the final analysis and prevent false significance. Removal of these overly influential observations were specifically done for MMSE (2 observations: scores of 6 and 2 at study exit), CDR-SB (1 observation: score of 18 at study exit), Digit Symbol (3 observations: scores of 83 and 88 at study entry, score of 7 at study exit), and SDMT (1 observation: score of 1 at study exit). Due to the use of multiple outcomes, results were only considered significant at *p* < 0.05 after a two-level correction to the *p* values. Significance for a given statistic was required to first meet a false-positive rate of *α* = 0.05 while maintaining 80% discriminative power. The significance of this selected set was then further adjusted using the more stringent Holm-Sidak family-wise error rate (FWER) correction to correct for multiple comparisons. Final significance of a given model was based on these FWER adjusted *p* values. A three-way interaction between sex, *APOE*4 status, and anesthesia exposure was the main variable used to evaluate any longitudinal contrasts between subject sex, genetic factors and surgical/general anesthetic exposure and identify final moderating effects among the three covariates of interest.

## Results

### Study population

Out of a total of 1033 participants with known *APOE* status, there were 382 men and 651 women while 253 participants had surgery/GA after study enrollment (men *n* = 89, women *n* = 164). The mean follow-up after study enrollment was 7.3 years (SD = 4.7), and the mean follow-up after surgery/GA was 6.3 years (SD = 4.3). Demographics, past medical history characteristics, and cognitive outcomes at study entry for all participants are listed in Table 2 along with differences according to sex. For the entire cohort, including all participants regardless of surgical exposure status, women entered the studies older (81.0 vs 78.4 years, *p* < 0.001) while the men were significantly more educated (15.0 vs 13.9 years, *p* < 0.0001) and had an increased prevalence of obesity (63% vs 29%, p < 0.0001). Overall, men were more likely to be *APOE*4 carriers (35% vs 28% *p* = 0.008) although this sex disparity was not observed for subjects with eventual exposure to surgery/GA (*p* = 0.76). The analytic cohort was 86% white and 14% non-white (Black, Asian, or Native American). There was no difference in APOE4 carrier prevalence in non-white compared to white participants (32% vs 20%, *p* = 0.27). With regard to participant comorbidities, there was no difference in average CIRS score nor prevalence of diabetes between the two sexes. There was, however, an increased prevalence of hypertension (74% vs 52%, *p* < 0.001) and thyroid disorders (11 vs 2%, *p* < 0.01) in the women exposed to surgery/GA when compared to men. Cognitive outcomes saw some sex distinctions at study entry with women showing better performance on MMSE (25.8 vs 26.7, *p* = 0.008), the CERAD delayed word list (5.4 vs 6.0, *p* = 0.002), and Digit Symbol test (34.9 vs 38.6, *p* = 0.003) while men exhibited better baseline performance on Animal Category Fluency (17.8 vs 16.3, p < 0.001).

**Table 2:** Cohort demographics and cognitive outcomes at study entry

	Non-surgical men (*n* = 297)	Non-surgical women (*n* = 493)	Surgical men (*n* = 89)	Surgical women (*n* = 164)	Sex effect *p* value	Surgical effect *p* value	APOE4 effect *p* value
Mean age, years (SD)	78.5 (10.3)	81.5 (9.2)	78.1 (7.8)	79.4 (7.7)	< 0.001	0.035	< 0.001
Mean education, years (SD)	14.8 (3.3)	13.7 (2.9)	15.9 (2.7)	14.5 (2.7)	< 0.0001	< 0.001	0.72
Cumulative Illness Rating Scale (SD)	22.5 (4.3)	22.4 (3.9)	21.8 (3.7)	21.6 (3.4)	0.66	0.003	0.002
Presence of an *APOE*4 allele (%)	39	28	22	25	0.008	0.014	–
Asian	0	6	1	0	–	–	–
Black	6	36	6	28	–	–	–
Native American	1	2	0	0	–	–	–
Caucasian/White	285	443	82	135	–	–	–
Obesity (%)	66	26	65	29	< 0.0001	0.51	0.065
Diabetes (%)	10	11	13	12	1	0.68	0.003
Hypertension (%)	56	65	52	74	< 0.001	0.014	0.082
Thyroid disorder (%)	2	7	2	11	< 0.0001	0.19	0.87
MMSE	24.9 (5.8)	26.0 (5.3)	28.6 (1.4)	28.5 (1.5)	0.008	< 0.001	< 0.001
CDR-SB	1.8 (2.9)	1.4 (3.1)	0.1 (0.27)	0.1 (0.26)	0.11	< 0.001	< 0.001
Logical Memory Delayed Recall	8.3 (4.7)	9.3 (4.9)	11.6 (4.3)	11.5 (4.2)	0.19	< 0.001	0.004
CERAD Delayed Word List	4.9 (2.4)	5.7 (2.5)	6.6 (1.7)	6.8 (2.0)	0.002	< 0.001	< 0.001
Digit Symbol	32.5 (13.6)	37.2 (10.6)	40.1 (8.8)	41.7 (11.4)	0.003	< 0.001	0.073
SDMT	39.9 (11.1)	38.3 (10.3)	47.2 (14.2)	39.7 (12.6)	0.088	0.40	0.62
Trails Making Test B	141.7 (80.8)	133.0 (69.2)	90.5 (30.1)	112.5 (45.6)	0.94	0.006	0.27
Animal Category Fluency	16.9 (5.3)	15.9 (5.2)	20.6 (5.0)	17.7 (4.7)	< 0.0001	< 0.001	0.30

### No sex differences in postoperative cognitive decline after exposure to surgery & general anesthesia

After controlling for age, level of education, *APOE4*, Cumulative Illness Rating Scale (CIRS) score, and baseline outcome values, there were many outcomes with altered rates of change after surgical exposure when pooling men and women together and comparing all exposed participants to all unexposed participants (Table 3, surgical exposure main effect). However, there were no sex differences in rate of postoperative cognitive decline after exposure to surgery/GA on any of the specific tests (Table 3, sex effect-surgical exposure). Further, there were no sex differences in the rate of decline in cognitive test scores when comparing men and women who were not exposed to surgery/GA (Table 3, sex effect-no surgical exposure).

**Table 3:** Rates of change in cognitive test scores in men vs. women

		Non-surgical men (slope)	Non-surgical women (slope)	Surgical men (slope)	Surgical women (slope)	Surgical exposure main effect (*p* value)	Sex effect—no surgical exposure (*p* value)	Sex effect—surgical exposure (*p* value)
Global cognitive function tests	MMSE	− 0.02528	− 0.01907	− 0.03539	− 0.02704	< 0.001	0.13	0.33
	CDR-SB	0.3568	0.2991	0.483	0.425	< 0.001	0.24	0.95
Memory-based tests	Logical Memory Delayed Recall	− 0.07798	− 0.08284	− 0.2623	− 0.2914	< 0.001	0.86	0.84
	CERAD Delayed Word List	−0.03858	− 0.0445	0.05887	− 0.05463	< 0.001	0.35	0.13
Attention-based tests	Digit Symbol	− 0.8686	− 0.948	− 1.244	− 1.271	< 0.001	0.31	0.65
	SDMT	− 1.312	− 0.8192	− 1.226	− 1.134	0.24	0.08	0.74
Executive function tests	Trails Making Test B	5.1	3.419	10.81	9.089	0.12	0.36	0.86
	Animal Category Fluency	− 0.4655	− 0.3809	− 0.5711	− 0.5551	< 0.001	0.15	0.35

### Older men with *APOE4*+ are at higher risk of postoperative cognitive dysfunction

**Fig. 1:**
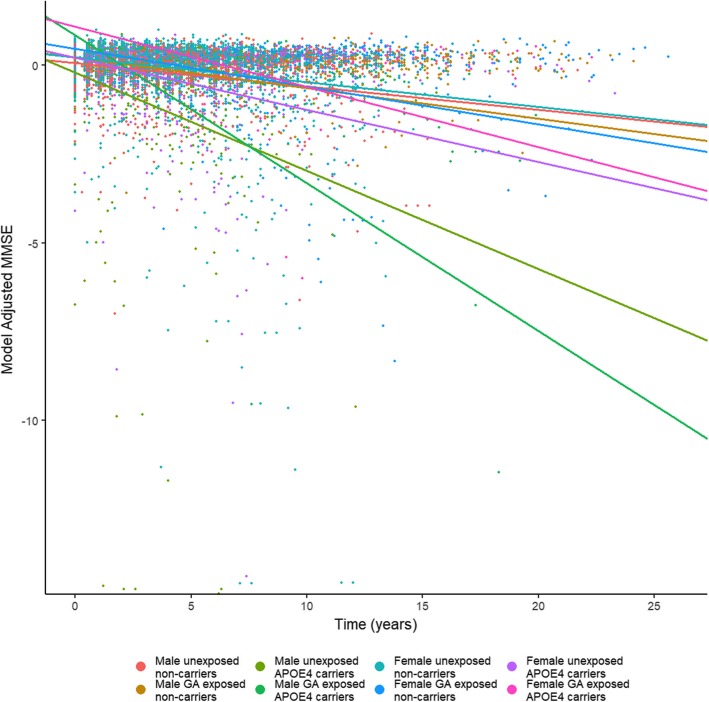
Rate of change in MMSE score. Rate of change scatterplot and group trajectories in model-adjusted MMSE score over the study period for each of the eight sex/genotype/surgical exposure groups. Time “0” corresponds to study enrollment. Each point represents an individual visit. MMSE Mini-Mental State Examination

Next, we divided the above cohort by *APOE4* carrier status, and repeated the analyses comparing the associations of surgery/GA exposure and sex within the same *APOE4* carrier to identify the difference in sex-exposure effects with respect to carrier status. The only outcome revealing a sex difference among *APOE4* non-carriers in rate of decline was CDR-SB: *APOE4*− women declined more rapidly after surgery in CDR-SB than *APOE4−* men (Table 4). For all other outcomes analyzed, there was no interaction between sex and surgical exposure for *APOE4* non-carriers. However, when we examined the sex-exposure interaction for *APOE4*+ carriers, we found that men with at least one copy of the *APOE4* allele who were exposed to surgery/GA had a more rapid rate of decline in tests of global cognitive function (MMSE and CDR-SB) and memory (Logical Memory, CERAD word list) (Fig. 1 and Table 5). There were no significant differences in decline on tests of attention or executive function between the two groups. (Tables 4 and 5).

**Table 4:** Rates of change in postoperative cognitive test scores in men vs. women stratified by *APOE4* status—*APOE4−* carriers

		Men *APOE4−* non-surgical (slope) *n* = 177	Women *APOE4−* non-surgical (slope) *n* = 348	Men *APOE4−* surgical (slope) *n* = 69	Women *APOE4−* surgical (slope) *n* = 123	Surgical-sex interaction on rate of change—Non-Carriers (*p* value)
Global cognitive function tests	MMSE	− 0.01485	− 0.01571	− 0.01948	− 0.02411	0.15
	CDR-SB	0.2749	0.2654169	0.3305	0.3888	*0.02*
Memory-based tests	Logical Memory Delayed Recall	− 0.05689	− 0.05369	− 0.1959	− 0.2873	0.19
	CERAD Delayed Word List	− 0.03427	− 0.04447	− 0.04921	− 0.05608	0.64
Attention-based tests	Digit Symbol	− 0.9422	− 0.9187	− 1.297	− 1.232	0.78
	SDMT	− 0.03789	− 0.02705	− 0.02942	− 0.03062	0.21
Executive function tests	Trails Making Test B	7.005	3.892	3.388	6.532	0.96
	Animal Category Fluency	− 0.4282	− 0.3635	− 0.5056	− 0.5563	0.15

*NS* not significant, italicized *p* values are significant

**Table 5:** Rates of change in postoperative cognitive test scores in men vs. women stratified by *APOE4* status—*APOE4+* carriers

		Men *APOE4*+ non-surgical (slope) *n* = 116	Women *APOE4*+ non-surgical (slope) *n* = 139	Men *APOE4*+ surgical (slope) *n* = 20	Women *APOE4*+ surgical (slope) *n* = 41	Surgical-sex interaction on rate of change—carriers (*p* value)
Global cognitive function tests	MMSE	− 0.0664115	− 0.0328237	− 0.09733	− 0.03718	*< 0.001*
	CDR-SB	0.6476	0.4505	1.059	0.581	*< 0.001*
Memory-based tests	Logical Memory Delayed Recall	− 0.1557	− 0.2307	− 0.5671	− 0.2724	*0.01*
	CERAD Delayed Word List	− 0.05731	− 0.04418	− 0.1063	− 0.0451	*0.03*
Attention-based tests	Digit Symbol	− 0.4937	− 1.074	− 1.07	− 1.467	0.65
	SDMT	− 0.1192	− 0.05094	− 0.1983	− 0.08353	0.91
Executive function tests	Trails Making Test B	7.437	3.938	6.532	4.561	0.84
	Animal Category Fluency	− 0.6281	− 0.4697	−0.8696	− 0.5259	0.31

*I*talicized *p* values are significant

## Discussion

Because not every older adult develops POCD following surgery and anesthesia, we explored whether biological risk factors were involved. Our findings demonstrated novel sex-*APOE4* allele interactions for postoperative cognitive decline. Similar to previous reports, we found that men and women had similar rates of postoperative cognitive decline [36–38]. However, sex differences emerged with stratified analyses, comparing women and men with the same *APOE4* carrier status. Men with an *APOE4* allele had more severe postoperative cognitive decline compared to women with an *APOE4* allele. These findings suggest a potential explanation for the discordance in the literature regarding the relationship between postoperative cognitive decline and the *APOE4* allele [16].

There has been increasing evidence that the presence of an *APOE4* allele confers disease risk in a sex-dependent manner. Studies have shown that *APOE4* confers a greater AD risk in women compared to men [17]. Women with *APOE4* also have an excess of AD pathology, including neuritic plaques and neurofibrillary tangles [39, 40]. Furthermore, at advanced ages such as those under study in this cohort, a much greater proportion of women are demented compared to men. We speculate that these factors create a “ceiling effect” in women such that the effects of additional brain insults in APOE4 females are not very potent, whereas in men there is more potential for surgery/GA to result in an increase in dementia. Additionally, there is complexity to the relationship between sex and *APOE4* carrier status. It has also been demonstrated that men who were *APOE4* carriers diagnosed with mild cognitive impairment (MCI) or AD have higher risks of brain microbleeds compared to *APOE4*-carrying women with MCI or AD [41].

The novel sex-A*POE4* allele interactions for postoperative cognitive decline suggest that *APOE4* carrier status may play an essential role in the male excess of postoperative delirium. While several studies have shown greater postoperative delirium among men [18–20], to date there are no investigations revealing a sex difference in postoperative cognitive dysfunction, though none of these investigations have assessed sex-*APOE4* interactions. Future studies could consider sex-genetic risk factor interactions in the incidence of deleterious postoperative neurocognitive outcomes, such as delirium and POCD.

Building knowledge regarding the potential impact of sex and *APOE4* genotype in older adults facing decisions regarding surgery is important for several reasons. First, if health care providers knew who was at a higher risk for postoperative cognitive decline, they would be able to more accurately counsel these individuals and their families regarding surgical risks and benefits, potentially leading to a decision to forego an elective surgical procedure. Second, this information would also inform key postoperative care and appropriate discharge planning. Many studies have shown that multidisciplinary proactive, personalized discharge planning contributes to increased patient satisfaction, decreased hospital length of stay, and reduced readmission to the hospital [42, 43].

One limitation of this investigation is that the surgical and anesthetic history was obtained by self-report from participants and/or caregivers. However, participants are interviewed twice annually in an attempt to accurately capture this type of health information, increasing confidence in this data. Because we do not have access to surgical or anesthetic records and are unable to account for potentially important differences including duration of general anesthesia, specific anesthetic agents used, or perioperative complications. Another limitation of this and other POCD studies is that participants undergoing surgery and anesthesia might have fundamental differences compared to those who did not have a surgical exposure. We did adjust for age, education level, and CIRS in our data analyses in an attempt to decrease this confounder. Finally, we note that although we had 1069 total participants in this study, only 57 participants that were exposed to surgery/GA were *APOE4+.* Despite this small number, we were still adequately powered to determine a significant rate of cognitive decline between groups.

## Conclusions

Using a retrospective analysis of multiple prospective, longitudinal aging studies, OBAS, ISAAC, KEAP, AADAPt, CBDP, OLL, and the Layton Alzheimer’s Disease Center patient registry, we have shown that *APOE4*+ men have an increased rate of decline in cognition after exposure to surgery and anesthesia than women *APOE4* carriers. This study builds upon prior research indicating that *APOE4* has sex-based health implications that may affect treatments. This study is an important initial step to begin to elucidate the role of sex and genetic variables on postoperative outcomes. Moving forward, researchers should consider testing for the effects of genes implicated in postoperative cognitive decline by sex, rather than combing data for both sexes. These investigations are necessary to move toward personalized strategies to improve postoperative outcomes in older adults.

## Abbreviations

AADAPt: African American Dementia and Aging Project
AD: Alzheimer’s disease
*APOE*: Apolipoprotein E
*APOE*4: Apolipoprotein E-ε4
Aβ: Amyloid β
CBDP: Oregon Community Brain Donor Program
CDR: Clinical Dementia Rating
CDR-SB: Clinical Dementia Rating sum of boxes
CERAD: Consortium to Establish a Registry for Alzheimer’s Disease
CIRS: Cumulative Illness Rating Score
FWER: Family-wise error rate
GA: General anesthesia
ISAAC: Intelligent Systems for Assessment of Aging Changes
KEAP: Klamath Exceptional Aging Project
MCI: Mild cognitive impairment
MMSE: Mini-Mental State Examination
OADC: Oregon Alzheimer’s Disease Center
OBAS: Oregon Brain Aging Study
OLL: Oregon Living Laboratory
POCD: Postoperative cognitive dysfunction
SD: Standard deviation
SDMT: Symbol Digit Modalities Test
